# Lateral Flow Device *Aspergillus* Routine Testing for Invasive Pulmonary Aspergillosis in Patients Who Are Critically Ill: A Multicenter Intensive Care Unit Cohort Study

**DOI:** 10.1093/ofid/ofaf256

**Published:** 2025-04-29

**Authors:** Stefan Hatzl, Lisa Kriegl, Christina Geiger, Philipp Eller, Robert Krause

**Affiliations:** Intensive Care Unit, Department of Internal Medicine, Medical University of Graz, Graz, Austria; BioTechMed Graz, Graz, Austria; BioTechMed Graz, Graz, Austria; Division of Infectious Diseases, Department of Internal Medicine, Medical University of Graz, Graz, Austria; Division of Infectious Diseases, Department of Internal Medicine, Medical University of Graz, Graz, Austria; Intensive Care Unit, Department of Internal Medicine, Medical University of Graz, Graz, Austria; BioTechMed Graz, Graz, Austria; Division of Infectious Diseases, Department of Internal Medicine, Medical University of Graz, Graz, Austria

**Keywords:** bedside testing, ICU, IPA, lateral flow device, rapid testing

## Abstract

**Background:**

The incidence of invasive pulmonary aspergillosis (IPA) is rising among intensive care unit (ICU) patients, with early diagnosis and treatment being critical for survival. Lateral flow assays for *Aspergillus* antigen detection have recently been introduced, enabling rapid results within an hour and potentially supporting earlier clinical decision making and timely antifungal therapy.

**Methods:**

This retrospective multicenter study included 180 ICU patients, 48 with IPA and 132 controls, across 9 treatment centers. Fungal infections were classified according to the FUNDICU criteria (Invasive Fungal Diseases in Adult Patients in Intensive Care Unit).

**Results:**

Among the 180 patients, 48 were classified as having probable IPA, while 132 exhibited host factors and radiologic findings consistent with IPA but did not meet the FUNDICU criteria. In this cohort, the sensitivity and specificity of the bronchoalveolar lavage lateral flow device test for diagnosing probable IPA vs no IPA were 71% (95% CI, 56%–83%) and 98% (94%–100%), respectively. The area under the receiver operating characteristic curve was 0.84, indicating good diagnostic performance. The positive and negative likelihood ratios were 31.17 (10.03–96.80) and 0.30 (.19–.46), yielding a diagnostic odds ratio of 104 (30–360). The positive and negative predictive values were 92% (78%–98%) and 90% (84%–95%).

**Conclusions:**

Lateral flow device testing may serve as a valuable tool for the rapid diagnosis of IPA in time-critical ICU settings. However, it is not sufficient to definitively rule out the disease, and a comprehensive diagnostic approach remains essential.

## Graphical Abstract

**Graphical Abstract:**
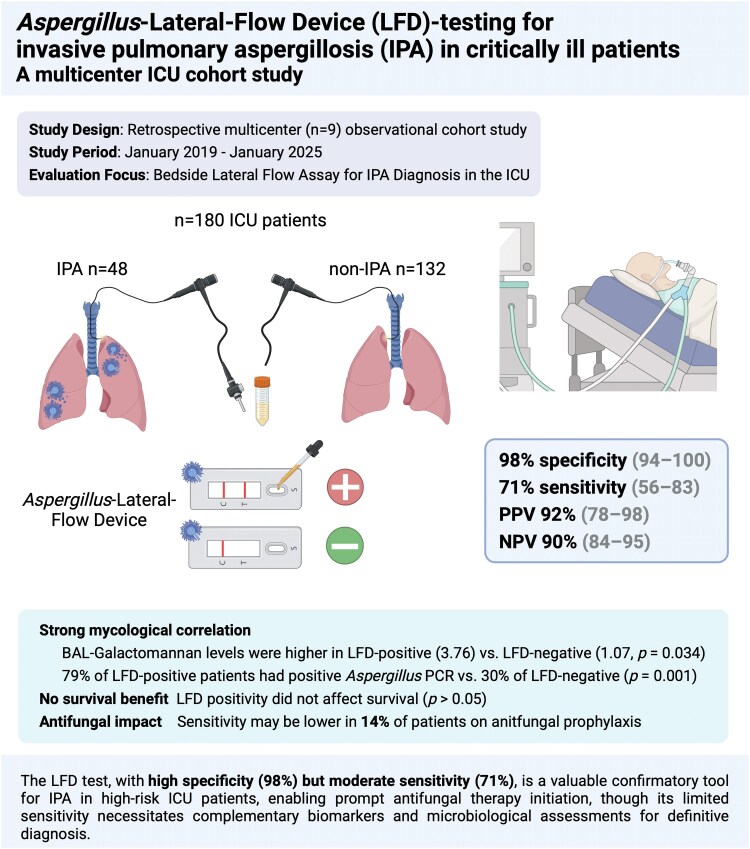
This diagram summarizes a retrospective multicenter observational study conducted from January 2019 to January 2025, including 9 treatment centers. The study focused on the use of bedside *Aspergillus* LFD testing for IPA in the intensive care unit (ICU). The study included 180 ICU patients at risk for IPA per the recent FUNDICU algorithm. Results showed that the *Aspergillus* LFD test had a specificity of 98%, sensitivity of 71%, positive predictive value (PPV) of 92%, and negative predictive value (NPV) of 90%. The study concluded that the *Aspergillus* LFD test is a valuable, easy-to-use, and rapid diagnostic tool for detecting IPA in ICU patients.

## Teaser

This multicenter study evaluated a bedside lateral flow device test for invasive pulmonary aspergillosis in 180 patients in the intensive care unit (48 cases, 132 controls). The test demonstrated 71% sensitivity and 98% specificity, showing good diagnostic accuracy and practical use in real-world settings.

Invasive pulmonary aspergillosis (IPA), caused by the ubiquitous mold *Aspergillus*, is a major cause of morbidity and mortality among patients in the intensive care unit (ICU) who are immunocompromised and nonneutropenic [1]. Adults who are nonneutropenic and critically ill with varying comorbidities represent a diverse group at risk for invasive fungal infections. To address this shift in the at-risk population, the FUNDICU algorithm (Invasive Fungal Diseases in Adult Patients in Intensive Care Unit) for diagnosing IPA in the ICU was recently developed [2]. The FUNDICU algorithm was created to standardize outcome definitions in clinical research on fungal diseases in the ICU setting. An initial validation study showed that the FUNDICU criteria outperform previously published diagnostic algorithms for IPA in clinical settings [1, 3, 4]. Galactomannan (GM) detection in serum or bronchoalveolar lavage fluid (BALF) remains the primary diagnostic method for IPA, alongside culture-based identification of *Aspergillus* species [2, 5]. GM detection has also been validated for noninvasive upper airway samples, highlighting its utility in different clinical settings [6]. The GM enzyme-linked immunosorbent assay has a turnaround time of approximately 3 hours and requires skilled laboratory personnel. However, it is typically performed in batches due to processing constraints, limiting its availability around the clock and reducing its utility for patients who are critically ill and require rapid decision making [7]. This is particularly concerning for patients with severe influenza, who may develop IPA upon ICU admission, where delays in initiating effective antifungal therapy are linked to higher mortality [8, 9]. Lateral flow assays for detection of *Aspergillus* antigen have recently been introduced, offering rapid testing within an hour, which may enable faster decision making and timely initiation of antifungal treatment [7, 10]. In this multicenter cohort study, we aimed to evaluate the diagnostic properties of an easy-to-use lateral flow assay for diagnosing IPA at the bedside in the ICU.

## METHODS

### Study Cohort

We conducted a multicenter observational study across 9 clinical centers in Austria, including patients admitted to the ICU from 1 January 2019 to 1 January 2025. *Aspergillus* LFD analysis was performed alongside conventional GM testing during the clinical workup (Supplementary Figure 1). The cohort included 48 consecutive adult patients diagnosed with IPA based on the FUNDICU algorithm, forming the case group. The control group consisted of 132 patients who had FUNDICU host factors and thoracic computed tomography changes consistent with the algorithm but tested negative for invasive fungal infection [2]. Patients with immunosuppression were classified according to the revised criteria of the European Organization for Research and Treatment of Cancer/Mycoses Study Group (EORTC/MSG), as outlined in the FUNDICU algorithm [11]. The study was approved by the local review board (EK: 32-302ex19/20) and conducted in accordance with the Declaration of Helsinki principles.

### Lateral Flow Assay Performance

We used an *Aspergillus*-specific lateral flow device (LFD; OLM Diagnostics), which detects an extracellular glycoprotein antigen secreted during active growth of *Aspergillus* spp. For routine LFD testing, 100 μL of untreated BALF samples were applied to the LFD. Results were visually assessed after a 15-minute incubation at room temperature, as recommended. Bound antigen-antibody-gold complexes were indicated by a red line, with intensity proportional to the antigen concentration. Test line intensity ranged from strong positive to weak positive or negative [12, 13]. OLM Diagnostics recently merged with IMMY, and the OLM LFD test is no longer available.

### Statistical Analysis

Statistical analysis was performed in R version 4.0.5 (https://www.r-project.org/). The diagnostic performance of the LFD for probable IPA vs no IPA based on the FUNDICU algorithm was evaluated. Proven IPA cases were diagnosed postmortem via necropsies whenever possible. Negative predictive value, positive predictive value, sensitivity, and specificity were calculated. Additionally, diagnostic odds ratios with 95% CIs were determined. Survival outcomes between patients who were *Aspergillus* LFD positive and negative were assessed in patients with probable IPA via Kaplan-Meier estimators. In addition, we investigated the impact of other *Aspergillus* biomarkers (bronchoalveolar lavage [BAL]–GM, serum-GM, *Aspergillus* polymerase chain reaction, and positive *Aspergillus* culture result) on 30-day overall survival in Cox regression models. To evaluate potential factors associated with false-negative LFD test results, we employed an unbiased approach by determining univariable predictors of false-negative LFD within logistic regression models and using adaptive LASSO regression (least absolute shrinkage and selection operator), including all baseline parameters to identify independent factors.

## RESULTS

### Study Cohort

A total of 180 adult patients admitted to the ICUs of our 9 treatment centers were evaluated for IPA via the FUNDICU algorithm, with *Aspergillus* LFD testing also available. LFD testing was conducted as part of the routine patient workup alongside other fungal parameters. Of these, 48 patients exhibited probable IPA, while 132 had host factors and radiologic findings consistent with IPA but did not meet the FUNDICU criteria and were thus classified as not having IPA. Respiratory samples for GM and LFD testing were collected via bronchoscopy. Of the 34 patients who were *Aspergillus* LFD positive, 33 had a strong reaction and 1 moderate. None showed a weak reaction.

The median age of the cohort was 63 years (IQR, 55–75), with patients with IPA being younger than controls at 61 years (49–69) vs 65 (55–75, *P* = .014). Among the cohort, 53 patients (29%) were female. The groups had similar immunosuppression profiles, as indicated by baseline blood counts, and approximately 30% of patients in both groups were classified according to the EORTC/MSG criteria due to underlying immunosuppressive conditions. Antimold prophylaxis use was comparable between groups, with 7 (14%) in the IPA group and 23 (17%) in the control group.

Regarding nonneutropenic ICU conditions, differences emerged between the groups. Patients with IPA more frequently had chronic obstructive pulmonary disease, while COVID-19 was more prevalent in the control group. Both groups were characterized by severe respiratory failure, but extracorporeal membrane oxygenation was more commonly used in patients with IPA than in controls. Detailed patient characteristics are summarized in Table 1.

**Table 1.** Baseline Characteristics of Cohort

	No. (%) or Median (IQR)			
Variable	Overall (n = 180)	IPA (n = 48)	No-IPA (n = 132)	*P* Value
Age, y	63 (55–74)	61 (49–69)	65 (55–75)	.014
Female	53 (29)	9 (18)	44 (33)	.058
Body mass index, kg/m^2^	27.9 (24.7–32.4)	25.6 (22.8–28.1)	28.6 (25.4–33.2)	.001
Laboratory findings				
Leukocytes, G/L	9.5 (6.8–13.9)	9.9 (6.9–15.9)	9.4 (6.8–13.2)	.552
Neutrophils, G/L	8.6 (5.9–12.1)	9.4 (5.8–13.7)	8.4 (5.9–11.7)	.661
Lymphocytes, G/L	0.7 (0.4–0.9)	0.6 (0.3–0.9)	0.7 (0.5–0.9)	.204
Hemoglobin, g/dL	11.5 (8.6–13.5)	9.4 (8.6–11.0)	12.3(10.7–13.8)	<.001
Platelets, G/L	207 (66–290)	133 (65–227)	221 (157–309)	<.001
CRP, mg/L	123 (71–191)	140 (88–217)	112 (70–177)	.075
Bilirubin, mg/dL	0.5 (0.4–0.9)	1.2 (0.5–2.3)	0.5 (0.4–0.7)	<.001
Creatinine, mg/dL	1.1 (0.8–2.0)	1.6 (0.9–7.0)	1.1 (0.8–1.4)	<.001
Host factor				<.001
EORTC/MSG risk factor	57 (32)	15 (31)	42 (32)	
Hematologic malignancy	28 (49)	7 (47)	21 (50)	
Allogeneic SCT	9 (16)	4 (27)	5 (12)	
Solid organ transplant	12 (21)	4 (27)	8 (19)	
Glucocorticoids	39 (68)	11 (73)	28 (66)	
Neutropenia	…	7 (46)	20 (47)	
B-cell inhibitor	7 (12)	3 (20)	4 (10)	
T-cell inhibitor	17 (30)	6 (40)	11 (26)	
Inborn error of immunity	1 (2)	1 (7)	0 (0)	
COVID-19	63 (35)	10 (21)	53 (40)	
Influenza	25 (14)	5 (10)	20 (15)	
Solid tumor	8 (4)	2 (4)	6 (5)	
Decompensated cirrhosis	7 (3)	4 (9)	3 (2)	
Moderate/severe COPD	20 (11)	12 (25)	8 (6)	
Fungal prophylaxis	20 (11)	7 (14)	23 (17)	.651
ICU characteristics				
SOFA	7 (4–9)	7 (4–8)	7 (4–9)	.614
PaO_2_/FIO_2_	125 (79–271)	125 (81–350)	136 (79–178)	.572
Ventilatory support				.001
Noninvasive ventilation	…	3 (6)	2 (1)	
Invasive ventilation	…	37 (77)	124 (94)	
vv-ECMO	…	8 (17)	6 (5)	

There were no missing data for either group.

Abbreviations: COPD, chronic obstructive pulmonary disease; CRP, C-reactive protein; EORTC/MSG, European Organization for Research and Treatment of Cancer/Mycoses Study Group; FIO_2_, fraction of inspired oxygen; ICU, intensive care unit; IPA, invasive pulmonary aspergillosis; SCT, stem cell transplantation; SOFA, sequential organ failure assessment; vv-ECMO, venovenous extracorporeal membrane oxygenation.

### Fungal Characteristics of the Patients With IPA

As outlined previously, 48 patients had probable IPA according to the FUNDICU criteria and, for patients treated in the ICU who were immunocompromised, the EORTC/MSG criteria. The most common *Aspergillus* species cultured from BALF was *Aspergillus fumigatus*, identified in 24 of 48 cases (50%). Interestingly, patients with a positive *Aspergillus* LFD result tended to have higher levels of mycologic biomarkers, particularly those obtained from BALF. BAL-GM levels were significantly higher in patients who were LFD positive (3.76; IQR, 2.16–7.45) as compared with patients who were LFD negative (1.07; IQR, 0.8–4.73; *P* = .034). Additionally, patients who were LFD positive had a higher frequency of positive *Aspergillus* polymerase chain reaction results from BALF at 19 of 24 (79%) vs 3 of 10 (30%) in patients who were LFD negative (*P* = .001). This finding was consistent for positive *Aspergillus* culture results overall, with 23 of 29 (79%) in patients who were LFD positive as compared with 6 of 29 (21%) in patients who were LFD negative (*P* = .042; Table 2). Despite this higher evidence of *Aspergillus* infection in patients who were LFD positive, a positive LFD result did not influence patient survival outcomes (Supplementary Figure 2). In addition, we analyzed the impact of other *Aspergillus* biomarkers on the 30-day survival in patients with IPA. Interestingly, none were associated with survival outcomes (Supplementary Table 1).

**Table 2.** Mycologic Findings of the Cohort

	No. (%) or Median (IQR)				
Mycologic Findings	Missing	Overall (n = 48)	*Aspergillus* LFD Negative (n = 14)	*Aspergillus* LFD Positive (n = 34)	*P* Value
Optical density index					
Serum-GM	0 (0)	0.32 (0.11–0.95)	0.12 (0.10–0.91)	0.32 (0.14–1.6)	.316
BAL-GM	0 (0)	3.58 (1.13–7.29)	1.07 (0.8–4.73)	3.76 (2.16–7.45)	**.034**
β-D-glucan, pg/mL	0 (0)	92.5 (15.4–254.7)	92.4 (15.4–485.0)	92.5 (29.4–235.0)	.937
*Aspergillus* PCR positive	14 (30)	24/34 (71)	3/10 (30)	19/24 (80)	**.001**
BAL culture positive	0 (0)	29 (60)	6 (20)	23 (80)	**.042**
* A fumigatus*		24 (85)	3 (50)	21 (92)	
* A flavus*		1 (3)	1 (16)	0 (0)	
* A calidostus*		1 (3)	0 (0)	1 (4)	
* A niger*		1 (3)	0 (0)	1 (4)	
* A terreus*		2 (6)	2 (33)	0 (0)	

Bold indicates *P* < .05.

Abbreviations: BAL, bronchoalveolar lavage; GM, galactomannan; LFD, lateral flow device; PCR, polymerase chain reaction.

### Diagnostic Properties of the *Aspergillus* LFD Test

**Figure 1.**
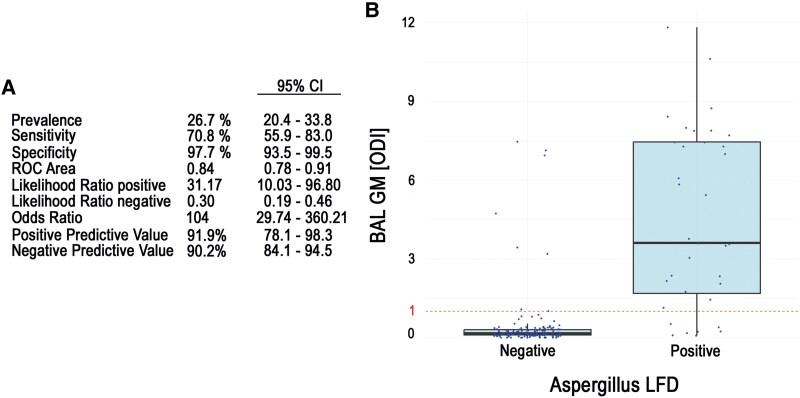
*A*, The diagnostic properties of the *Aspergillus* lateral flow device (LFD) test. *B*, The box plots of bronchoalveolar lavage–galactomannan (BAL-GM) tests in relation to a positive or negative *Aspergillus* LFD test result. Data are presented as median (line), IQR (box), and 95% CI (error bars). ODI, optical density index; ROC, receiver operating characteristic curve.

The sensitivity and specificity of the BAL LFD test for diagnosing probable IPA vs no IPA in our cohort were 71% (95% CI, 56%–83%) and 98% (94%–100%), respectively, when applied to patients with FUNDICU or EORTC/MSG host factors and radiologic findings consistent with the criteria. The area under the receiver operating characteristic curve for the LFD test was 0.84. The positive and negative likelihood ratios were 31.17 (10.03–96.80) and 0.30 (.19–.46). The diagnostic odds ratio for IPA was 104 (30–360). The positive and negative predictive values were 92% (78%–98%) and 90% (84%–95%). For the LFD test as a surrogate for established mycologic factors, the sensitivity for positive BAL-GM and *Aspergillus* culture was calculated. The sensitivity of the BAL LFD test for a positive BAL-GM test result (optical density index >1) was 71% (56%–83%; 30 of 39 patients had positive LFD results). The LFD test had a sensitivity of 80% for a positive *Aspergillus* culture finding (23 of 29 patients with a positive culture finding had a positive LFD result). Diagnostic properties are summarized in Figure 1. Of the 48 patients diagnosed with IPA, 32 died, and 18 of these underwent necropsy with histopathologic examination. Among the 18 necropsies, 15 showed histopathologic evidence of proven IPA.

The sensitivity and specificity of the *Aspergillus* LFD for diagnosing postmortem proven IPA were 92% (95% CI, 62%–100%) and 83% (36%–100%), respectively. The area under the receiver operating characteristic curve was 0.88, indicating good overall diagnostic performance. The positive likelihood ratio was 5.5 (.91–33.18), and the negative likelihood ratio was 0.10 (.01–.68), resulting in a diagnostic odds ratio of 55.00. The positive and negative predictive values were 92% (62%–100%) and 83% (36%–100%).

### Risk Factors Associated With False-Negative LFD Test Results

Having demonstrated high specificity in our ICU cohort, which consisted of patients with a high pretest probability for IPA but lower sensitivity, we sought to determine whether independent factors were associated with false-negative results. First, we used logistic regression models to analyze potential associations between false-negative LFD results and baseline parameters. We found that high absolute leukocyte counts and belonging to the EORTC/MSG host factor group were associated with lower odds of false-negative *Aspergillus* LFD results (Supplementary Table 2). To identify independent predictors of false-negative LFD results, we applied an adaptive LASSO regression as an unbiased approach suitable for small event numbers (Supplementary Figure 3). Interestingly, none of the baseline characteristics were associated with false-negative LFD results.

## DISCUSSION

We report the utility of a bedside *Aspergillus* LFD in the ICU, acknowledging the recent shift in IPA classification toward patients who are critically ill and nonneutropenic as an at-risk population. Our analysis focused on a cohort of patients with a high pretest probability of IPA, including those who met the host criteria and radiologic findings defined by the recent FUNDICU algorithm [1, 2]. This is reflected by an exceptionally high prevalence of 26.7%. The composition of our cohort is important to note, as there are differences in host factors between patients with IPA and the control group of patients without IPA. For example, more patients with COVID-19 in the control group underwent LFD testing than those in the IPA group. This likely reflects early findings of COVID-19–associated pulmonary aspergillosis, which prompted more aggressive diagnostics for invasive fungal diseases in these patients as compared with others, resulting in a higher number of patients without IPA in the control group [14, 15]. *Aspergillus* LFD positivity serves as a surrogate marker for mold growth in the tracheobronchial system, which explains the association with higher BAL-GM levels and successful culture of *Aspergillus* spp. The observed correlation between LFD positivity and the BAL biomarker but not the serum biomarker likely reflects the higher diagnostic sensitivity of BAL as compared with serum [16]. Ultimately, since LFD positivity indicates the presence of *Aspergillus* spp in the tracheobronchial tree, this correlation is to be expected. Using the JF5 monoclonal antibody, the LFD test in this study detects an extracellular mannoprotein antigen secreted during the active growth of *Aspergillus* species. Several biological factors, including environmental growth conditions (eg, pH and carbon source due to viral replication), can influence the amount of mannoprotein and other fungus-specific proteins released, potentially affecting the binding capacity of the JF5 antibody [12]. In addition, host-related factors such as prior or ongoing antifungal therapy may affect LFD sensitivity, as this has been demonstrated in large clinical studies and in animal models for other fungal proteins [17]. Bedside testing for IPA is an attractive approach for the early identification of patients with this critical condition, particularly in an ICU setting where timely treatment is crucial [9]. We also investigated the impact of *Aspergillus* LFD positivity alongside other established biomarkers on 30-day survival in patients with IPA. Surprisingly, none of these biomarkers significantly affected survival. This may be due to the varying host factors in our cohort, known to be major determinants of survival, and the limited sample size. For example, serum-GM, a marker of angioinvasion, showed a trend toward worse outcomes, which aligns with previous reports [18]. However, these findings are exploratory and require validation in larger studies. A major difference between our cohort and previous reports on bedside LFD testing for IPA is that the testing in our study was conducted during routine patient care, with BALF analyzed immediately rather than being stored and tested later [7, 10, 19, 20]. As expected for a population in which the test is used as a confirmatory tool for patients judged to have a high probability of IPA, the LFD demonstrated very high specificity of nearly 98% and moderate sensitivity of 71%. As with any diagnostic algorithm, including the FUNDICU criteria, there is an inherent diagnostic “gray zone” that introduces a degree of uncertainty [1]. This makes such algorithms challenging to define as a true gold standard, as the only definitive proof of invasive fungal disease remains the histopathologic demonstration of invasive hyphae in tissue. To address this, we evaluated the diagnostic performance of the LFD against postmortem proven cases of IPA. The LFD demonstrated a sensitivity of 92% and a specificity of 83%, thereby confirming its diagnostic accuracy when compared with histologic evidence. This finding suggests that the LFD could be utilized as a tool to confirm the presence of IPA and promptly initiate antimold therapy. However, the LFD alone is not sufficient to completely rule out IPA. These findings represent the classic trade-off of single-parameter tests. Future research could minimize this trade-off in sensitivity by combining the LFD with risk scores for IPA in the ICU setting [21, 22]. The moderate sensitivity might also be influenced by antifungal prophylaxis, which was applied in 14% of patients with IPA, thus lowering the sensitivity of mycologic biomarker testing [10].

Easy-to-use bedside tests such as the LFD may serve as a practical surrogate for BAL-GM diagnostics, particularly in time-sensitive ICU settings where comprehensive laboratory workups may be limited, such as nights or weekends. In such cases, a positive LFD result could support the diagnostic algorithm in place of BAL-GM, potentially enabling the timely initiation of mold-active treatment in patients with suspected IPA. However, given the lack of large-scale clinical trials validating rapid testing systems, IPA diagnoses should still be reevaluated by conventional methods and established biomarkers, as outlined in diagnostic frameworks such as the FUNDICU criteria.

Therefore, we conclude that LFD testing could serve as an important tool for quickly diagnosing IPA in time-critical ICU situations. However, it is not sufficient to fully rule out IPA, and a comprehensive workup including biomarkers and microbiological techniques remains essential.

## Supplementary Material

ofaf256_Supplementary_Data

## Supplementary Data

Supplementary materials are available at *Open Forum Infectious Diseases* online. Consisting of data provided by the authors to benefit the reader, the posted materials are not copyedited and are the sole responsibility of the authors, so questions or comments should be addressed to the corresponding author.

## Notes

***Author contributions.*** S. H. and R. K. designed the study. S. H. wrote the first draft. S. H. performed the biometry. S. H. and R. K. interpreted the results. S. H. and R. K. collected the data. All authors read and revised the manuscript.

***Data availability.*** The data set used and analyzed during the current study is available from the first author on reasonable request.
