# Supplementary material for: Lateral Flow Device Aspergillus Routine Testing for Invasive Pulmonary Aspergillosis in Patients Who Are Critically Ill: A Multicenter Intensive Care Unit Cohort Study
Source: Open Forum Infect Dis. 2025 Apr 29;12(5):ofaf256. doi: 10.1093/ofid/ofaf256 (PMC12086331; doi:10.1093/ofid/ofaf256)
Supplement: ofaf256_Supplementary_Data [file ofaf256_supplementary_data.docx]

**Supplementary –** **Lateral flow-device aspergillus routine -testing for invasive pulmonary aspergillosis in critically ill patients – A multicenter ICU cohort study**

# Hatzl S. et al.

#
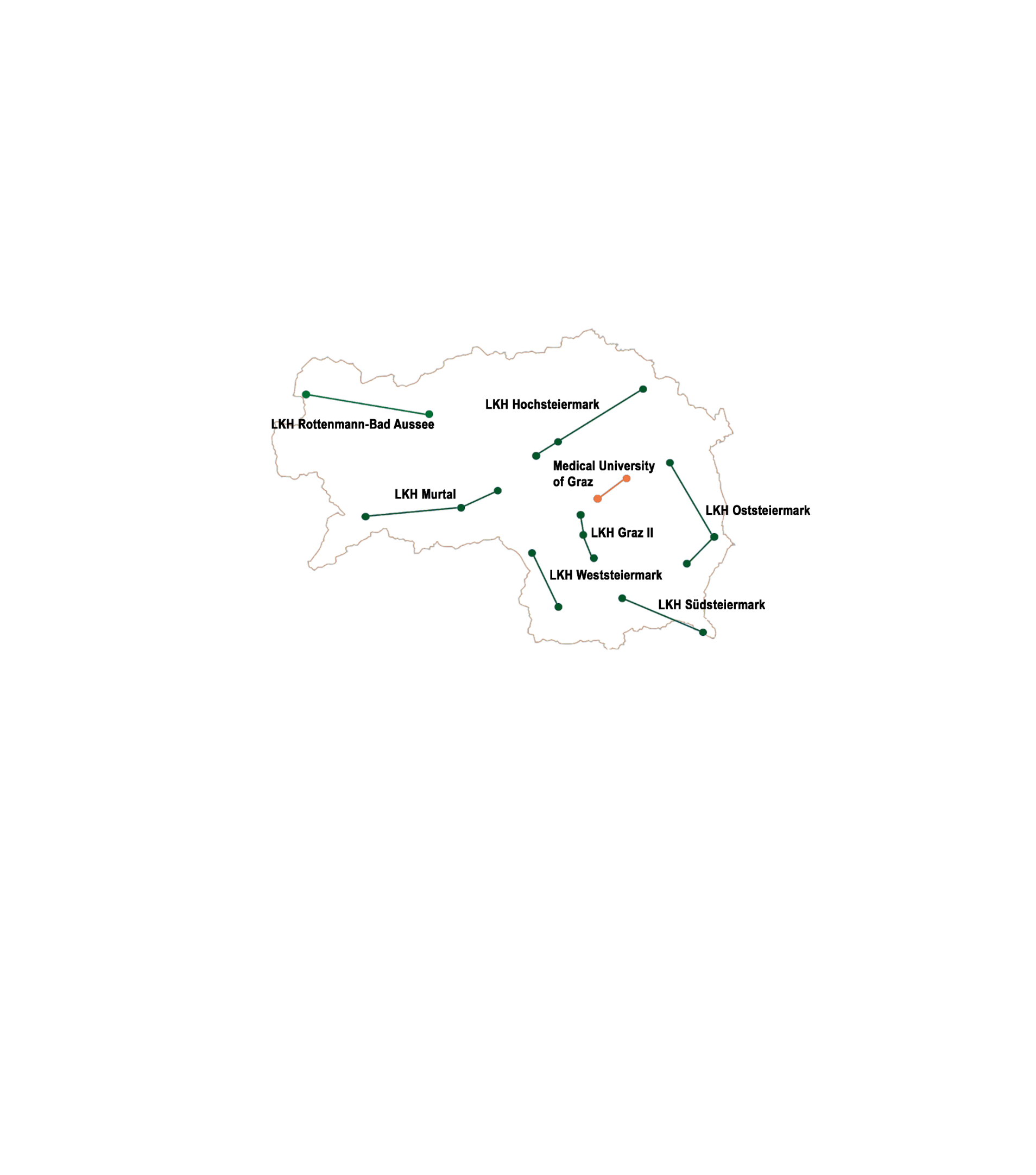


| **Center** | **n** |
| --- | --- |
| LKH-Graz (Internal Medicine) | 110 |
| LKH-Graz (Surgery) | 40 |
| LKH-Graz (Neurology) | 3 |
| LKH-Oststeiermark (Mixed) | 5 |
| LKH-Südsteiermark (Mixed) | 3 |
| LKH-Weststeiermark (Mixed) | 3 |
| LKH-Hochsteiermark (Mixed) | 5 |
| LKH-Graz II (Mixed) | 9 |
| LKH-Rottenmann/ Bad Aussee ((Mixed)) | 2 |

**Supplementary Figure 1: Treatment centers involved in the study**

Each point in the figure refers to a single hospital. The connecting lines show the hospital-networks. LKH – hospital network, n= number of participants enrolled by each center


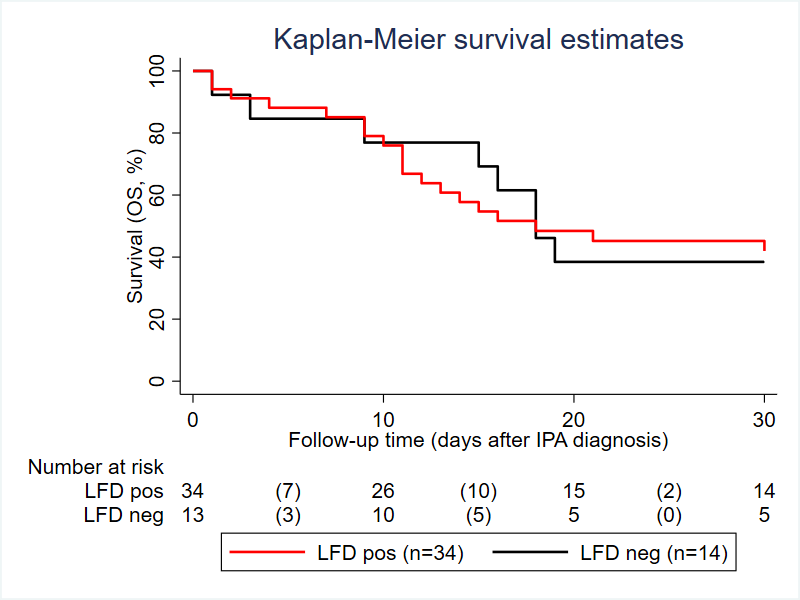


**Supplementary Figure 2: Survival of patients with and without a positive LFD was analyzed using Kaplan-Meier estimators**

LFD – lateral flow device


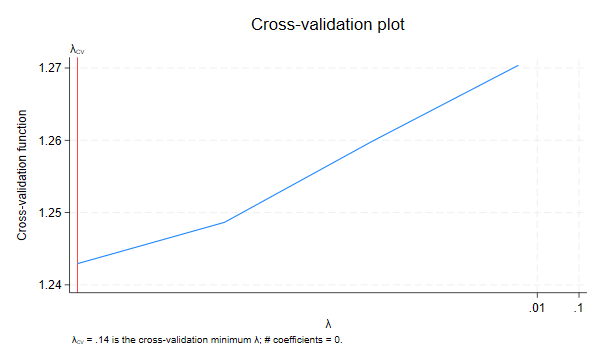


**Supplementary Figure 3: Cross-validation plot for adaptive LASSO regression.**

The cross-validation plot shows that no coefficients were associated with false-negative *Aspergillus*- LFD-results.

LFD – lateral flow device; LASSO – least absolute shrinkage and selection operator

| **Variable** | **HR [95%-CI]** | **p-value** |
| --- | --- | --- |
| BAL-GM | 1.02 [0.90-1.16] | 0.69 |
| Serum-GM | 1.18 [0.97-1.43] | 0.08 |
| *Aspergillus* culture | 0.77 [0.46-1.28] | 0.33 |
| *Aspergillus* PCR | 0.65 [0.31-1.39] | 0.27 |

**Supplementary Table 1: Univariable Cox-regression of Aspergillus biomarker for 30-day OS**

None of the established Aspergillus biomarkers showed an association with 30-day overall survival in Cox models. HR – hazard ratio; GM – galactomannan; PCR – polymerase chain reaction; CI -confidence interval, OS – overall survival

| **Variable** | **OR [95%CI]** | **p-value** |
| --- | --- | --- |
| Age per 5 years increase | 0.98 [0.95-1.02] | 0.54 |
| Female Gender | 4.00 [0.45-35.48] | 0.21 |
| BMI per 1 kg/m² increase | 0.90 [0.77-1.04] | 0.16 |
|  |  |  |
| **Laboratory findings** |  |  |
| Leukocytes per 1 G/L | 0.88 [0.79-0.99] | **0.04** |
| Neutrophils per 1 G/L | 0.88 [0.78-1.00] | 0.06 |
| Lymphocytes per 1 G/L | 0.21 [0.04-1.07] | 0.06 |
| Hemoglobin per 1 g/dL | 0.85 [0.61-1.20] | 0.37 |
| Platelets per 50 G/L | 1.01 [0.79-1.29] | 0.89 |
| CRP per 100 mg/L | 0.74 [0.38-1.42] | 0.37 |
| Bilirubin per 1 mg/dL increase | 1.21 [0.92-1.61] | 0.17 |
| Creatinine per 1 mg/dL increase | 0.90 [0.72-1.13] | 0.38 |
|  |  |  |
| **Host factor** |  |  |
| EORTC/MSG risk factor | 0.19 [0.05-0.74] | **0.02** |
| COVID-19 | 1.42 [0.21-9.58] | 0.71 |
| Influenza | N/A |  |
| Solid Tumor | N/A |  |
| Decompensated Cirrhosis | N/A |  |
| Moderate/ Severe COPD | N/A |  |
|  |  |  |
| **Fungal prophylaxis** | 4.13 [0.78-21.69] | 0.09 |
|  |  |  |
| **ICU-Characteristics** |  |  |
| SOFA per 1 point increase | 0.99 [0.88-1.17] | 0.97 |
| paO_2_/FiO_2_ per 50 units increase | 1.50 [0.88-2.55] | 0.132 |

**Supplementary Table 2: Univariable predictors of false-negative LFD-results**

The univariable odds-ratio were derived from logistic regression models. N/A states that the corresponding variable was not calculatable. LFD- lateral flow device; BMI – body mass index; CRP – C-reactive protein; EORTC-MSG - European organization for research and treatment of cancer – Myosis Study Group; COVID-19 – coronavirus disease-19; COPD – chronic obstructive pulmonary disease; SOFA – sequential organ failure assessment
